# Recent advances in self-assembling peptide matrices as functional coatings for implantable devices

**DOI:** 10.3389/fchem.2022.1040499

**Published:** 2022-11-16

**Authors:** Yuan Tian, Lei Lu

**Affiliations:** ^1^ Sichuan Engineering Research Center for Biomimetic Synthesis of Natural Drugs, School of Life Science and Engineering, Southwest Jiaotong University, Chengdu, China; ^2^ School and Hospital of Stomatology, Wenzhou Medical University, Wenzhou, China

**Keywords:** self-assembling peptides, functional coatings, surface modification, implantable devices, nanofiber

## Abstract

Nature has evolved numerous supramolecular machineries for modulating various cellular functions. Inspired by the assembly of these sophisticated structures in nature, the controlled assembly of synthetic peptides emerges as a promising approach to therapeutically relevant applications. The self-assembling biomimetic peptides could form well-ordered architectures through non-covalent interactions such as π-π stacking, van der Waals, electrostatic, and hydrogen bonding. In addition, the peptidic building blocks are highly biocompatible and allow facile chemical manipulation with diverse functionalities. For decades, a serious of engineered self-assembling peptides have been extensively studied as functional hydrogels for various applications. Meanwhile, the surface modification strategies based on self-assembling peptide matrices have also raised the attention of biomaterials researchers due to their programmability and 3D porous morphologies. This concise review will cover recent advances in self-assembling peptide matrices as functional coatings for implantable devices. The opportunities and challenges in this field will also be discussed.

## Introduction

Peptide self-assembly has emerged as an attractive strategy for achieving diverse supramolecular structures for therapeutically relevant applications (Wang et al., 2016; Levin et al., 2020). These naturally existing building blocks are short segments of proteins, which possess well-established chemistry, chemical diversity, and biocompatibility. Due to the structural simplicity of the amino acids, peptides can be readily designed and synthesized for a range of applications to realize the same functionalities as proteins. Inspired by the assembly of sophisticated structures in nature, the spontaneous arrangements of peptide molecules into well-ordered nanostructures can be achieved through noncovalent interactions, such as π-π stacking, van der Waals, electrostatic and hydrogen bonding, which is considered one of the most versatile materials in biomedical engineering (Sinha et al., 2021).

The past decades have witnessed rapid advances in biotechnology and material science to develop numerous implantable devices such as drug-eluting stents in vascular intervention, scaffolds for tissue engineering, and orthopedic/dental implants (Lin et al., 2013). Nevertheless, their biocompatibility remains a critical issue in limiting *in vivo* and clinical performance, particularly the inflammatory responses and potential foreign body reactions induced by the implantable devices. As a result, functional coatings of implantable devices have been vigorously studied to conquer the potential risks to patients (Kastellorizios et al., 2015).

This concise review will cover recent advances in self-assembling peptide matrices as functional coatings for implantable devices. The opportunities and challenges in this field will be discussed as well.

## Self-assembling peptides

### Peptide amphiphiles

**FIGURE 1:**
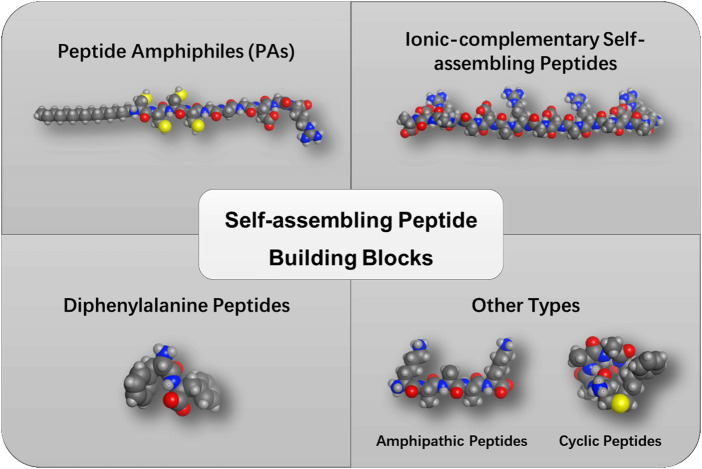
Categories of self-assembling peptide building blocks.

Peptide amphiphiles (PAs) constitute an important category of self-assembling peptides with enormous biological functionalities, which are featuring with a hydrophobic segment such as lipids at the termini (Figure 1). In 2001, Stupp et al. reported the first example of self-assembling PAs that formed hydrogel to mimic the extracellular matrix (Hartgerink et al., 2001). The lipidated peptide with an alkyl tail was found to possess a high β-sheet forming propensity, followed by arranging to form nanostructures. Since this discovery, the same group continued to study how to control the self-assembly of PAs by modulating the peptide sequences, the alkyl tail, concentration, pH value, and salts. The PAs have been reported to form diverse structures including cylinders, ribbons, twisted structures, etc., and bioactive components like functional peptide motifs could be linked to the PAs to achieve the desired biomedical applications (Hendricks et al., 2017). For instance, PAs have been shown to induce the differentiation of progenitor cells into neurons by encapsulating neural progenitor cells within PA nanofibers functionalized with a laminin epitope IKVAV (Silva et al., 2004).

### Ionic-complementary self-assembling peptides

Inspired by the Z-DNA binding protein decades ago, a class of novel self-assembling peptides with a unique complementary arrangement of charged amino acids was discovered. Sequence analysis revealed a 16-mer peptide (AEAEAKAKAEAEAKAK) that could form β-sheets with ionic-complementarity properties (Zhang et al., 1993). After that, a rich number of self-assembling peptides derived from natural proteins or mimicking typical protein secondary structures have been reported. These ionic-complementary peptides can self-assemble into nanofiber networks and gels depending on their sequence, concentration, and pH value or the presence of salts. Another well-known ionic-complementary peptide is the (RADA)_4_ or RADA16 (RADARADARADARADA) peptide, which forms a regular nanofiber structure. Their physicochemical properties and biomedical applications have been extensively investigated so far (Gelain et al., 2020). These ionic-complementary peptides constitute an alternating arrangement of positively and negatively charged residues. These ordered charge patterns with unique electrostatic, hydrogen bonding, and van der Waals forces drive their molecular self-assembly.

### Diphenylalanine peptides

Diphenylalanine (FF), consisting of two phenylalanine residues, represents a minimalistic self-assembling building block derived from the core motif of pathogenic amyloid-β peptide (Xiong et al., 2019). Previous studies revealed that FF-based peptides can self-assemble into various nanomorphologies, such as vesicles, nanowires, and nanofibrils. The driving force for the self-assembly of FF-based peptides is the π–π stacking of aromatic residues as well as the hydrogen bonding of the peptide backbone. Furthermore, the self-assembly of FF-based peptides can be regulated and controlled by enzymes, thereby achieving enzyme-tuned self-assembly *in vitro* or *in vivo*. The formation of the hydrogels generally includes the bond formation of the hydrogelator or the removal of a masking group from the hydrogelator precursor. A rich number of enzymes have been reported so far, such as kinase, β-lactamase, or phosphatase (Yang et al., 2008).

### Other types

Amphipathic peptides have been reported to form supramolecular structures in water. One of the representative categories is the surfactant-like peptide which is composed of consecutive hydrophobic residues (alanine, valine, leucine) and one or two hydrophilic residues (aspartic acid, glutamic acid, lysine, histidine, arginine) in the peptide sequence (Zhao, 2009). Another type of amphipathic peptide is the bolaamphiphilic peptide, which consists of two hydrophilic head groups connected by a hydrophobic core. This double-headed design offers highly diverse morphologies by tuning the head groups and the core sequence of the peptides. For instance, the KAAAAK, KAAAAAAK, and RAAAAAAR bolaamphiphilic peptides can form amyloid-like aggregation. However, the EFLLLLFE forms peptide nanotubes at a certain concentration (Lee et al., 2021).

Despite the applications of linear peptides as supramolecular building blocks, cyclic peptides have also attracted considerable attention as a more stable peptidic self-assembly building block. One of the cyclic peptides was reported to form a β-sheet-like tubular structure through intermolecular hydrogen bonding. The backbone of the cyclic peptide is positioned to the inner side of the cylinder with the side chain residues positioned outside the cylinder (Scanlon and Aggeli, 2008). Li et al. designed the coiled-coil nanofibers from cyclic α-helical pentapeptides (Hu et al., 2018). In addition, constrained α-helices were also found to trigger the self-assembly. Gazit et al. reported the assemblies formed by aminoisobutyric acid stabilized α-helical heptad peptides (Mondal et al., 2015).

## Functional coatings based on self-assembling peptide matrices

**FIGURE 2:**
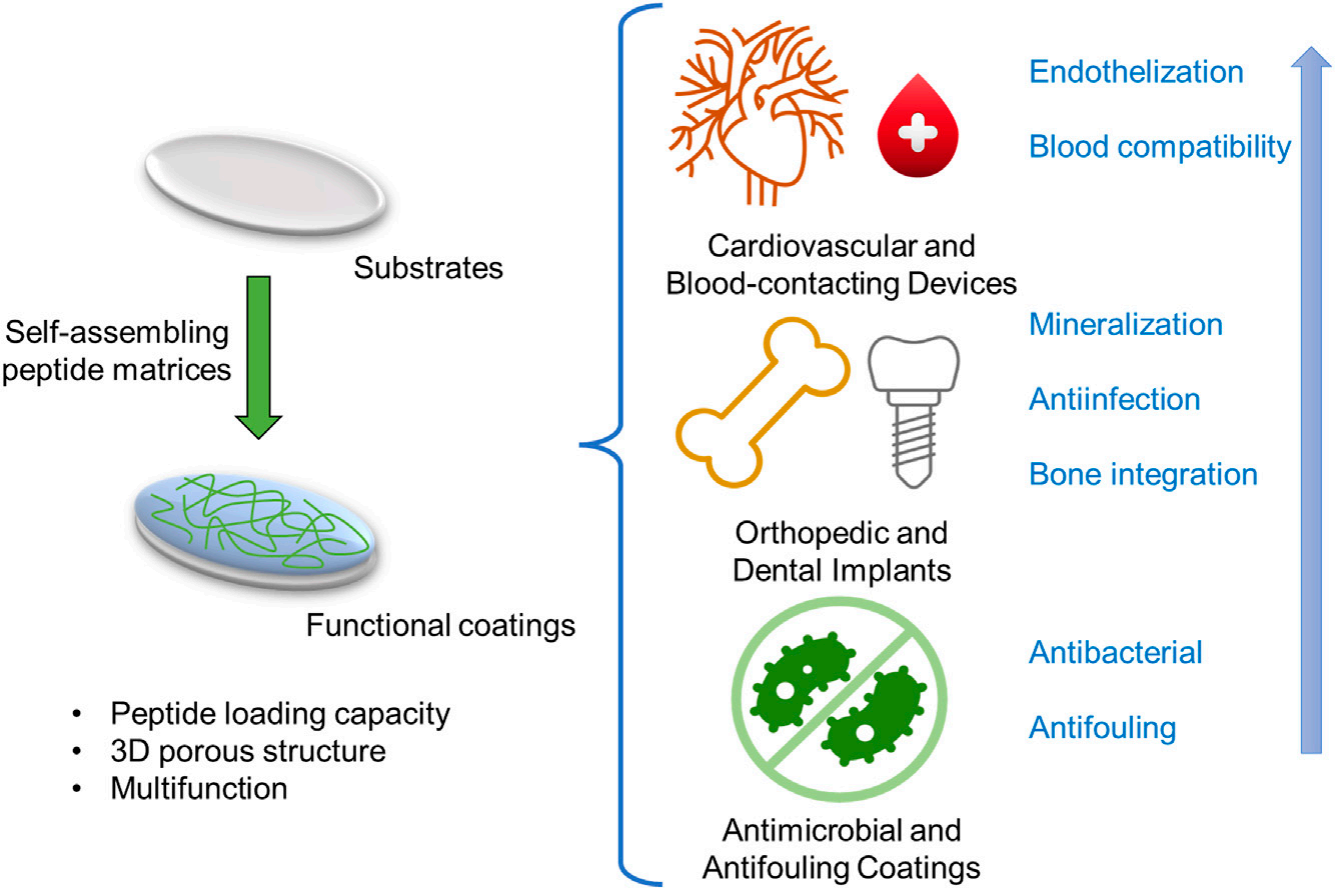
The illustration of self-assembling peptide-based functional coatings for implantable devices.

Functional peptides have been widely used in the surface modification of various biomaterials to enhance their biocompatibility. Conventional surface modification of medical implants by functional peptides is based on covalent interactions between peptide and surface. Compared to peptide grafting, the surface coating strategies based on self-assembling peptide matrices have apparent advantages in peptide loading capacity, 3D porous structure, multifunction, and relatively simple fabrication due to their materials’ nature. For decades, several self-assembling peptide-based surface functionalization techniques have been investigated for improving the biocompatibility of implantable materials, including cardiovascular and blood-contacting devices, orthopedic and dental implants, and other implants with antimicrobial and antifouling coatings (Figure 2 and Table 1).

**TABLE 1:** Self-assembling peptide matrices as functional coatings for implantable devices.

Substrates	Types	Functional ligands	Effects	References
Cardiovascular and Blood-contacting Devices				
316L SS	(RADA)_4_	NO-releasing catalyst: U	Antithrombotic ability *in vitro* and *ex vivo*	Li et al. (2022)
316L SS	PAs	NO donating: KKKKK; Endothelial cell adhesive: YIGSR; MMP-2 sensitive: GTAGLIGQ	Providing an endothelium mimicking environment	Kushwaha et al. (2010)
316L SS	PAs	NO donating: KKKKK; Endothelial cell adhesive: YIGSR; MMP-2 sensitive: GTAGLIGQ	Endothelium mimicking nano matrix coating for drug-eluting stents	Andukuri et al. (2014)
PCL nanofibers	PAs	Nitric oxide (NO) donating: KKKKK; Endothelial cell adhesive: YIGSR; MMP-2 sensitive: GTAGLIGQ	Increased adhesion and proliferation of endothelial cells, reduced smooth muscle cell proliferation, and reduced platelet adhesion	Andukuri et al. (2011)
316L SS	PAs	Surface adhesive: Dopa Selectively promote endothelial cell adhesion and spreading: REDV	Promote endothelial cell growth	Ceylan et al. (2011)
Au	FF	N/A	As an alternate system for polymer coating in drug-eluting stents	Zohrabi et al. (2016)
Orthopedic and Dental Implants				
TiAl6V4	PAs	Surface adhesive: Dopa Osteogenic: KRSR	Promote osteoblast-like cells and inhibited fibroblasts	Ceylan et al. (2012)
Au	GL13K	self-assembled antimicrobial amphiphiles: GL_13_K	Efficient self-assembled AMP coatings for dental and medical applications	Ye et al. (2020)
Ti	GL13K	self-assembled antimicrobial amphiphiles: GL_13_K	Against implant infections	Ye et al. (2022)
HA	EAbuK 16-II	N/A	Increasing the material bioactivity	Secchi et al. (2020)
Graphene foam	LLVFGAK	Mineralization: MLPHHGA	Promote biomimetic mineralization of HA.	Li et al. (2018)
DBM	(RADA)_4_	N/A	Promotes bone reconstruction	Hou et al. (2014)
Antimicrobial and Antifouling Coatings for Other Implants				
Ti	MIKA2	N/A	Inhibit colonization of Ti implants in mice	Fichman et al. (2021)
Waterborne PU	Fmoc-FF	PEG, PCL and K	Construct hydrophilic surface and hydrophobic subsurface	Zhang et al. (2018)
Si	Fmoc-FFY	N/A	Against Gram-positive bacterial infections	Criado-Gonzalez et al. (2020)
Si	VVD/LLE/VVE	Antifouling: PFB	Resist biofilm formation	Arul et al. (2020)
TiO_2_	EAK16-II	Antimicrobial: Chitosan	Support hNPs attachment and growth	Secchi et al. (2019)

### Cardiovascular and blood-contacting devices

Hemocompatibility is one of the major challenges for blood-contacting devices, which limits their clinical performance. Recently, we have reported active center selenocysteine (U) of glutathione peroxidase functionalized (RADA)_4_ hydrogel coating with the nitric oxide (NO)-generating activity for blood-contacting devices (316L stainless steel (316L SS) as an example) (Li et al., 2022). Moreover, the restenosis and incomplete endothelialization hindered the long-term clinical success of implantable cardiovascular devices, including stents and vascular grafts, etc. Endothelium mimicking self-assembling nanofiber matrix has been developed as a surface coating for a series of biomaterials and devices. The matrix is formed by PAs modified with NO donating residues (KKKKK), endothelial cell adhesive ligands (YIGSR), and matrix metalloproteinase-2 (MMP-2) cleavage sites (GTAGLIGQ) (Kushwaha et al., 2010; Andukuri et al., 2011; Andukuri et al., 2014). These nanofiber matrix-based coatings have successfully enhanced the growth of endothelial cells and inhibited both smooth muscle cell proliferation and platelet adhesion of cardiovascular implants and blood-contacting devices.

Dopa (3,4-dihydroxy phenyl-L-alanine) molecule is a biocompatible mussel-inspired adhesive structure for biomolecule immobilization on metal surfaces. The REDV motif selectively promotes the growth of endothelial cells. Nanofibers formed by Dopa- and REDV-conjugated PAs were used as a surface coating on 316L SS, which is widely used as the backbone of vascular stents (Ceylan et al., 2011).

Currently, the most used stent is a drug-eluting stent that contains a drug-eluting polymer coating on its surface. It has been reported that the diphenylalanine (FF) nanotubes can be used as an alternate drug-eluting system for polymer coating in stents (Zohrabi et al., 2016).

### Orthopedic and dental implants

Titanium (Ti) and its alloys-based orthopedic and dental implants are increasingly used in the treatment of bone defects and tooth loss due to their excellent corrosion resistance and good hard-tissue compatibility, which are commonly limited due to insufficient bone integration, and potential infections. PAs functionalized with adhesive Dopa and osteogenic peptide motif KRSR can be self-assembled as nanofiber coating on the surface of TiAl_6_V_4_ alloy, which creates an osteoconductive interface and inhibits the growth of fibroblasts (Ceylan et al., 2012). Antimicrobial coatings are promising strategies to prevent bacterial infections in implants, however, their long-term and *in vivo* performance is usually unsatisfactory. Self-assembly of the antimicrobial GL13K peptide-based supramolecular amphiphiles that can reduce bacterial infections (Ye et al., 2020). It has been reported that a hybrid nanocoating using self-assembled antimicrobial GL13K decorated with silver nanoparticles (AgNPs) increases antimicrobial potency against Ti implant infections (Ye et al., 2022).

Hydroxyapatite (HA) coatings are one of the conventional surface modification techniques for Ti alloys to improve biocompatibility and bioactivity. It has been reported that HA coatings can be further functionalized by EAbuK 16-II self-assembling peptide overlayer on their surface (Secchi et al., 2020).

A self-assembling peptide-mediated biomimetic strategy can also be adapted to the functionalization of 3D graphene foam with a motif-specific peptide (LLVFGAKMLPHHGA), which exhibits biomimetic mineralization ability. Such peptide sequence consists of two functional motifs: the β-sheet self-assembling LLVFGAK sequence and the biomimetic mineralization motif MLPHHGA (Li et al., 2018).

Additionally, (RADA)_4_ self-assembling peptide can build a cellular microenvironment that promotes bone reconstruction. The bone reconstruction of demineralized bone matrix (DBM) was enhanced by (RADA)_4_ nanofiber matrix which induced smaller pore size and stronger charge interaction (Hou et al., 2014).

### Antimicrobial and antifouling coatings

Other than orthopedic and dental implants, surface modification strategies based on antimicrobial peptides (AMPs) have been extensively developed for preventing bacterial infections in other biomedical implants.

As previously described by Dopa-mediated surface adhesion strategies, mussel-inspired adhesive molecular or materials design plays an important role in the surface modification of implantable biomaterials. Recently, it was demonstrated that a peptide derived from mussel foot protein, displays antibacterial properties, which inspired the design of self-assembling peptide (MIKA2) based adhesive hydrogels with antimicrobial activity against drug-resistant Gram-positive bacteria on Ti (Fichman et al., 2021).

Polyurethane (PU) based biomaterials have favorable mechanical properties and biocompatibility that have great potential in implant devices. It has been demonstrated that polyethylene glycol (PEG) motif functionalized Fmoc-diphenylalanine (Fmoc-FF) self-assembling peptide endows waterborne PU surfaces with enhanced biocompatibility, antifouling ability, and water resistance, simultaneously (Zhang et al., 2018).

Enzyme-triggered self-assembly of Fmoc-FF-based short peptides was developed. Fmoc-FFpY peptide (p: PO_4_^2–^), can be dephosphorylated into Fmoc-FFY and form an antibacterial hydrogel coating by previously deposited alkaline phosphatase functionalized NPs@AP nanoparticles on the surface. The resulting Fmoc-FFY peptides then self-assemble into β-sheets nanofiber hydrogel coating, which inhibits the growth of Gram-positive *Staphylococcus aureus* (*S. aureus*) (Criado-Gonzalez et al., 2020).

For antifouling coatings, two simple PAs functionalized with PFB (pentafluorobenzaldehyde), namely, PFB-VVD and PFB-LLE was designed and can self-assemble into functional coatings with anti-parallel β-sheet secondary conformation on the surface. As described, PFB-VVD exhibits better antifouling activity compared to PFB-LLE (Arul et al., 2020).

Chitosan grafted EAK16-II self-assembling peptides-based hydrogel coating on TiO_2_ surface, which has a protentional to support cell attachment and growth while against infections by the antimicrobial nature of chitosan. The deposited EAK16-II possesses layer-by-layer and ordered structures depending on the preparative stoichiometry and path (Secchi et al., 2019).

## Discussion

The utilization of peptide self-assembly to form well-ordered structures offers great potential in biomedical engineering. This concise review summarizes the most used peptide building blocks and their applications as self-assembled functional coatings for implantable devices. Despite the significant advances in peptide-based self-assembly nanomaterials. How to precisely control the assembly of the peptide and understand the mechanistic insight of the assembly process will be highly desired to drive the discovery of new peptidic building blocks’ superior properties in the future. Another concern regarding the self-assembling peptide is their stability issues for *in vivo* applications. Moreover, introducing surface adhesive elements or using intermediate coating for effective immobilization of nanostructures on the surface is essential for ensuring their long-term stability. Although challenges remain, the peptide self-assembly holds great promise to further broaden its applications in biology and nanotechnology.
